# Synthesis, Characterization, and Investigation of Novel Ionic Liquid-Based Tooth Bleaching Gels: A Step towards Safer and Cost-Effective Cosmetic Dentistry

**DOI:** 10.3390/molecules28073131

**Published:** 2023-03-31

**Authors:** Memuna Kausar Satti, Maleeha Nayyer, Meshal Alshamrani, Muhammad Kaleem, Ahmad Salawi, Awaji Y. Safhi, Abdullah Alsalhi, Fahad Y. Sabei, Abdul Samad Khan, Nawshad Muhammad

**Affiliations:** 1 Department of Dental Materials, National University of Medical Sciences (NUMS), Rawalpindi 46000, Pakistan; 2 Department of Pharmaceutics, College of Pharmacy, Jazan University, Jazan 45142, Saudi Arabia; 3 Department of Restorative Dental Sciences, College of Dentistry, Imam Abdulrahman Bin Faisal University, Dammam 34212, Saudi Arabia; 4 Department of Dental Materials, Institute of Basic Medical Sciences, Khyber Medical University, Peshawar 25100, Pakistan

**Keywords:** tooth bleaching gels, ionic liquids, tooth color, microhardness, surface profilometry

## Abstract

The objective of this study was to synthesize a novel choline hydroxide ionic liquid-based tooth bleaching gel. Ionic liquid-based gels were synthesized and characterized using FTIR along with pH testing. Tooth sample preparation was carried out in line with ISO 28399:2020. The effects of synthesized gels on tooth samples were tested. Tooth samples were stained and grouped into three experimental groups: EAI (22% choline hydroxide gel), EAII (44% choline hydroxide gel), and EB (choline citrate gel) and two control groups: CA (commercial at-home 16% carbamide peroxide gel) and CB (deionized water). The tooth color analysis, which included shade matching with the Vitapan shade guide (*n* = 2), and digital colorimetric analysis (*n* = 2) were evaluated. The surface characteristics and hardness were analyzed with 3D optical profilometry, Scanning Electron Microscopy (SEM), Energy Dispersive X-ray Spectroscopy (EDX), and Microhardness testing (*n* = 3), respectively. The tooth color analysis (Vitapan shade guide) revealed that all the tooth samples treated with synthesized choline citrate gel (EB) showed an A1 shade as compared to the other four groups, giving a range of shades. An analysis of the ΔE values from digital colorimetry; EAI, EAII, CA, and CB showed ΔE values in a range that was clinically perceptible at a glance. However, EB showed the highest value of ΔE. The mean microhardness values for the five groups showed that the effects of three experimental gels i.e., 44% choline hydroxide, 22% choline hydroxide, and choline citrate, on the microhardness of the tooth samples were similar to that of the positive control, which comprised commercial at-home 16% carbamide peroxide gel. SEM with EDX of three tested subgroups was closely related in surface profile, elemental composition, and Ca/P ratio. The roughness average values from optical profilometry of four tested subgroups lie within approximately a similar range, showing a statistically insignificant difference (*p* > 0.05) between the tested subgroups. The synthesized novel experimental tooth bleaching gels displayed similar tooth bleaching actions without any deleterious effects on the surface characteristics and microhardness of the treated tooth samples when compared with the commercial at-home tooth bleaching gel.

## 1. Introduction

Cosmetic dentistry has broadened its scope with the increasing demand for aesthetics. Tooth discoloration is caused due to changes in the enamel, dentin, or pulp of the tooth by a chromogen, affecting its light-transmitting properties [1]. Other causes of tooth discoloration can be genetics, certain diseases, medication, dental materials, tooth trauma, environment, poor dental hygiene, and aging [2]. The discoloration can have intrinsic and extrinsic causes, depending upon the origin and incorporation of the staining or discoloring component on/in the tooth surface, respectively [3]. Tooth bleaching is a noninvasive procedure employed to restore or improve the original color of teeth. It is used as a conservative option and sometimes as an auxiliary treatment option for color abnormalities [4]. There are in-office and at-home tooth bleaching procedures to restore or improve the appearance of teeth [2].

Vital tooth bleaching may lead to sensitivity and mucosal burns [5]. Moreover, studies on human and bovine teeth have revealed alterations in surface microhardness, surface morphology, and the composition of teeth [6,7].

Different materials are used under the umbrella of in-office and at-home bleaching procedures. The primary ones include hydrogen peroxide, which is mainly used for in-office procedures. In-office tooth bleaching generally involves two appointments of 30 min each with a gap of 7 days in between [8]. Hydrogen peroxide has been under research for carcinogenesis, protein damage, and its effect on the mineral content of teeth [9,10].

Carbamide peroxide is used as an active ingredient in at-home tooth bleaching. For this, the bleaching material is loaded in the tray, and the patient is prescribed to wear the tray with material for seven hours daily for two weeks [2]. Other agents that are used or under research include blue covarine (organic pigment in water/glycerine), peroxy caproic acid, alumina, calcium carbonate and silica particles, sodium perborate, baking soda, peroxides, phosphate salts, and enzymes [5,11].

Peroxide-based tooth bleaching agents are caustic to oral and dental tissues [12]. In clinical trials with 10% carbamide peroxide, irritation of the gingiva was reported [13]. The potential of bleached teeth to be stained again in a shorter duration has been reported [10]. Both high and low pH of bleaching products are intended to increase the efficacy of the process. However, it can be detrimental to the oral tissues, since this also goes against the 7.4 pH of the oral cavity, disturbing the overall homeostasis and damaging the organic and inorganic contents of the tooth [9].

Ionic liquids are purely ionic, salt-like compounds and remain liquid below 100 °C [14]. These innovative liquids are exploited as eco-friendly, organic, cost-effective materials to synthesize various chemicals [15]. Amongst many types, choline hydroxide and choline citrate are used in biomedical research for iron deficiency anemia and postoperative ileus, and these are reported to be biocompatible ionic liquids [16,17]. 1-n-butyl-3-methylimidazolium bis(trifluoromethanesulfonyl)imide (BMI.NTf2) microcapsules loaded novel resin infiltrant were prepared to manage non-cavitated carious lesions in proximal dental surfaces [18]. Garcia et al. reported that zinc-based particles with 1-*n*-butyl-3-methylimidazolium chloride ionic liquid without changing the physiochemical properties improved the antibacterial activity against *S. mutans* of the dental adhesive [19].

This in vitro research aimed to evaluate choline hydroxide and choline citrate as tooth bleaching materials in gel form, providing a cost-effective bleaching gel with equivalent bleaching action and surface characteristic alterations of teeth as compared to the conventionally used at-home carbamide peroxide gel. Commercial choline hydroxide (44%) and its diluent (22%) were mixed with starch to obtain their gels. Moreover, choline hydroxide (44%) was reacted with citric acid to obtain choline citrate (viscous gel). The effects of synthesized gels and conventionally used at-home carbamide peroxide gel on tooth samples were tested. A tooth color analysis, which included shade matching with the Vitapan shade guide (*n* = 2), and digital colorimetric analysis (*n* = 2) were performed. The surface characteristics and hardness were analyzed with 3D optical profilometry, Scanning Electron Microscopy (SEM), Energy Dispersive X-ray Spectroscopy (EDX), and microhardness testing (*n* = 3), respectively.

## 2. Methodology

### 2.1. Materials

All the materials used in this study were of analytical grade and obtained from Sigma Aldrich, St. Louis, MO, USA.

### 2.2. Synthesis of Choline Hydroxide-Based Gels

For 44% choline hydroxide-based gel preparation, 10 mL of choline hydroxide (44%, Sigma Aldrich) was heated at 50 °C for 30 min, followed by adding 0.15 g of starch and stirred for 2 h to obtain a clear, colorless gel. For the preparation of 22% choline hydroxide-based gel, 44% choline hydroxide was diluted with water, followed by adding starch to obtain the corresponding gel.

### 2.3. Synthesis of Choline Citrate Gel

The choline citrate was obtained by treating 14.5 mL of choline hydroxide (44%) with 9.6 g of citric acid contained in one neck flask connected with a reflux setup at 70 °C and 300 rpm magnetic stirring speed for 6 h. The solution was rotary evaporated to remove water and obtained a choline citrate clear, colorless gel.

### 2.4. Characterization of Synthesized Gels

#### 2.4.1. pH Measurement

The pH of 44% choline hydroxide, 22% choline hydroxide gel, 44% choline hydroxide gel, choline citrate gel, and commercial at-home carbamide peroxide (Perfecta 16% Whitening Gel, B0010DMNQU, Edinburgh, Scotland, UK) tooth bleaching gel were measured using a digital pH meter (HANNA, Leighton Buzzard, UK) and a semi-micro-pH electrode (ORION, Turku, Finland) at room temperature: 21 °C ± 2 °C after calibration with buffered solutions having pH 4 and 7 (Mallinckrodt Baker, Inc., Phillipsburg, NJ, USA). Three readings were taken, and then, an average value was computed.

#### 2.4.2. Fourier Transform Infrared Spectroscopy (FTIR)

Choline hydroxide, 22%, 44% choline hydroxide gel, and choline citrate gel were subjected to FTIR in ATR mode (Thermo Nicolet 6700, Thermo Fisher Scientific, Waltham, MA, USA). The range for the collection of spectra was 4000–400 cm^−1^, with a resolution of 4 cm^−1^.

### 2.5. Tooth Sample Preparation

Tooth sample preparation was done following ISO 28399:2020 (Products for External Tooth Bleaching). Ethical permission from the ethical review committee (ERC/ID/166) of the National University of Medical Sciences, Rawalpindi, was obtained.

#### 2.5.1. Collection of Tooth Samples

The tooth samples used for the study included bovine teeth. The permanent and sound teeth (The buccal surface was intact without wear, caries, or fracture, and the non-hypoplastic or non-fluorosis buccal surface of teeth) were extracted from cattle aged between 30 and 72 months. The age of the animal was determined according to the standard operating procedure of the Food Safety and Inspection Service (FSIS). The collected samples were disinfected with sodium hypochlorite and then washed with deionized water [20]. The samples were then stored in deionized water in the refrigerator at 4 °C.

#### 2.5.2. Cutting of Enamel Blocks

Sample preparation was done according to ISO 28399:2020 (Products for External Bleaching). The crown root portion was separated at the cementoenamel junction, and the coronal portion was used for sample preparation. Two samples per tooth were taken from its buccal aspect. A total of 112 samples was prepared with the dimensions of 2 mm × 3 mm × 8 mm using a Linear Precision Saw (ISOMET 4000, Buehler, Lake Bluff, IL, USA). A plus-shaped mark was made at the back of all the tooth samples with a straight fissure bur to recognize the buccal aspect of the tooth.

#### 2.5.3. Mounting and Polishing of Enamel Blocks

Samples were mounted in an impression compound (Kemdent, Swindon, UK) for polishing. All the samples were polished with 800 and 1200 grit silicon carbide sandpaper sheets, followed by 3, 2, 1, and 0.5 microns Al_2_O_3_ on an automatic lapping and polishing machine (EQ-UNIPOL-1502, Qingdao, China) at a speed of 127 rpm. The samples were then washed and sonicated in deionized water for 30 s to remove residual abrasives without affecting the structure.

### 2.6. Staining of Tooth Samples

The staining solution used in this study was black tea (Tapal Tea Pvt. Ltd., Karachi, Pakistan) in water boiled at 100 °C. The samples were dipped in staining solution and kept in a water bath at 37 °C for 24 h. After that, the samples were removed and rinsed with deionized water before analysis.

#### 2.6.1. Grouping for Bleaching Treatment of Tooth Samples

The distribution of the groups is tabulated in Table 1. The artificially stained tooth samples were then randomly distributed among these groups. The immersion in 5 mL of the respective gels was done for 7 h (1 night), 49 h (7 nights), and 98 h (14 nights) in duration. The assembly was kept in an incubator (Memmert GmbH, Büchenbach, Germany) at 37 °C.

#### 2.6.2. Tooth Color Analysis

For the shade evaluation of the tooth samples, ISO 28399:2020 specification was followed. The Vitapan 3D shade guide (Vita Zahnfabrik, Bad Säckingen, Germany) and a chromameter (Konica MINOLTA CL-200A, Tokyo, Japan) were used to evaluate the bleaching action of the experimental and control groups. Shade matching of the sample was done with the Vitapan classical shade guide with 16 shade guide teeth in a color matching booth (GTI ColorMatcher, GTI Graphic Technology, Newburgh, NY, USA) using D65 daylight (6500 K). The shade guide teeth were arranged according to value from the lightest to the darkest, according to the manufacturer’s instructions, on a white background. The best visually matched shade guide tooth with the target sample was selected, and the shade number was recorded. Sample size was *n* = 2 for each group.

### 2.7. Digital Colorimetry

To measure the color of a tooth, a Castor colorimeter (Nordmeditec GmbH) was used. This colorimeter has a 0-degree/0-degree illumination/observation and measures the reflected emission of spectral colors by the use of 512 light-sensitive diodes in a 0.7-mm-diameter area. The light reflected from the tooth is emitted by an intense light source integrated into the colorimeter. The calorimeter calculates the color parameters in the L*a*b* color space. The total color differences (ΔE) were calculated for the experimental and control groups according to the following formula:

ΔE = [(ΔL*) ^2^ + (Δa*) ^2^ + (Δb*) ^2^]^1/2^ (1)

The standard perception range for analysis of the ΔE value was outlined as

- 1–0: Not perceptible by the human eye.
- 1–2: Perceptible through close observation.
- 2–10: Perceptible at a glance.
- 11–49: Colors are more similar than the opposite.
- 100: Colors are exactly opposite.

#### 2.7.1. Microhardness

A Vickers microhardness tester (Wolpert, 401 mvd eqpt 0002, Berg Engineering, Berlin, Germany) was used. Vickers Hardness Numbers (VHN) for the samples (*n* = 3 for each group) were obtained at a load of 50 g for 15 s, with three readings on each sample.

#### 2.7.2. Scanning Electron Microscopy (SEM)

All tooth samples were processed appropriately, including dehydration and sputter coating with gold in a high-vacuum sputter coating machine (MED 5010, blazers Union Liechtenstein). The specimens were observed using a SEM (JSM-6490A, Jeol, Tokyo, Japan) at a voltage of 10 kV, whereby the magnifications ranged from 500× to 20,000×. Semi-quantitative Energy Dispersive X-ray Spectroscopy (EDX; EDAX microanalysis system, Octane Plus Silicon Drift Detector, “TEAM Enhanced” v. 4.3) was used for the elemental analysis of the tooth samples.

### 2.8. Optical Profilometry

The surface roughness of the samples was assessed by a 3D optical profilometer (SPM-9500J3, Shimadzu Corp. Japan, Kyoto, Japan). An optical scan was made with the probe at a clock speed of 1 s with frequencies 154 Hz and 175 Hz, probe tips 3–6 µm, heights 15–40 nm of the end radius, and a 20 × 20 µm scan area.

#### Statistical Analysis

The analysis was established by SPSS version 22 software (IBM Chicago, IL, USA). Categorical variables were presented as frequencies and percentages, while quantitative variables were presented by the means and standard deviation. When the results followed the normality curve, the comparison between groups was carried out by one-way analysis of variance (ANOVA) and post hoc Tukey’s test. A *p-*value ≤ 0.05 was considered statistically significant.

## 3. Results

### 3.1. pH Measurement

The mean pH values of the 44% choline hydroxide, 44% choline hydroxide gel, 22% choline hydroxide gel, and commercially used at-home tooth bleaching gel (control) are tabulated in Table 2. The mean pH ranged from 12.4 ± 0.15 (basic pH of 44% choline hydroxide liquid) to 5.53 ± 0.20 (acidic pH of 16% carbamide peroxide gel).

### 3.2. Fourier Transform Infrared Spectroscopy

**Figure 1:**
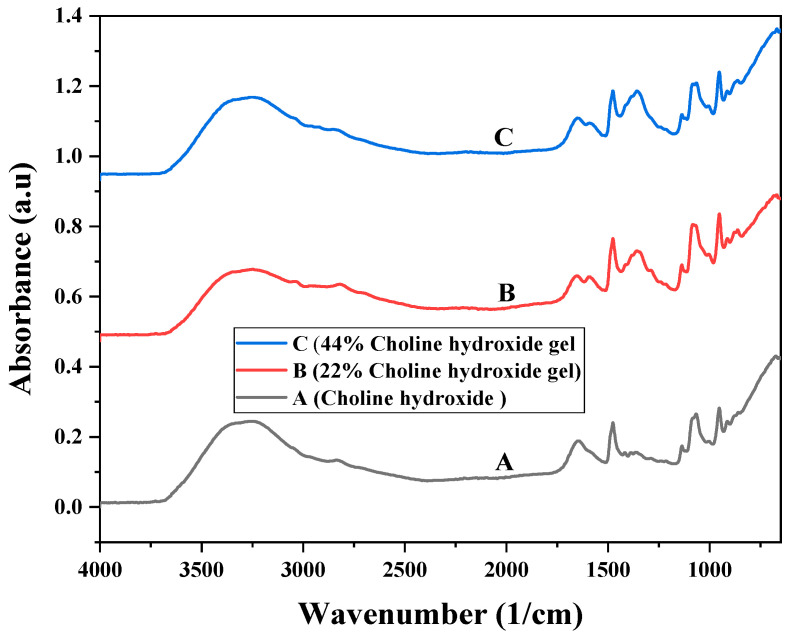
FTIR spectra of (A) 44% choline hydroxide ionic liquid, (B) 44% choline hydroxide ionic liquid gel, and (C) 22% choline hydroxide ionic liquid gel.

The FTIR spectra for 44% choline hydroxide, 22% choline hydroxide gel, and 44% choline hydroxide gel are shown in Figure 1. The O–H stretching vibration can be identified at 3600–3200 cm^−1^, the aliphatic C–H stretching vibration at 2800 cm^−1^, O–H bending at 1640 cm^−1^ and methyl deformation was witnessed at 1472 cm^1^. C–O stretching was observed at 1077 cm^1^. The deformation of the –CH2– group was detected around 951 cm^1^ [21,22]. The C–O–C stretching of starch observed at 1345 cm^1^ was not present in choline hydroxide.

**Figure 2:**
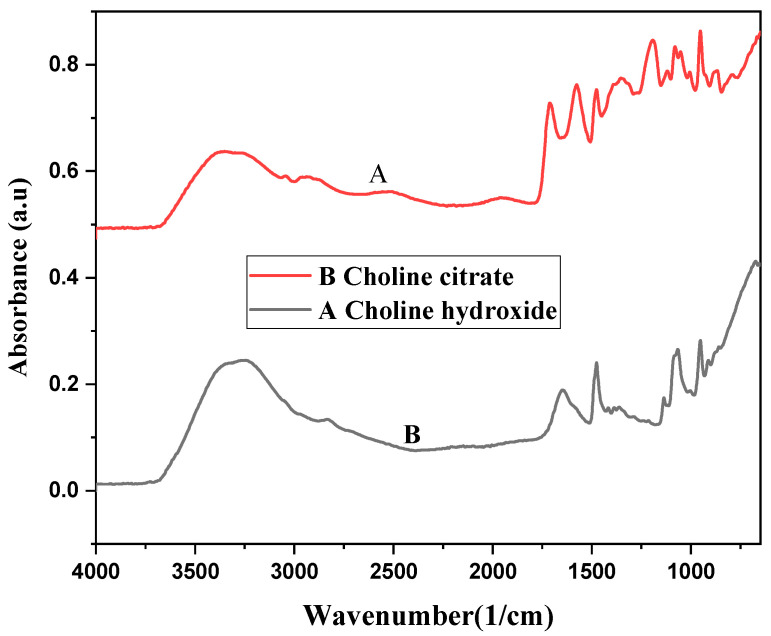
FTIR spectra of choline hydroxide (A) and choline citrate gels (B).

The FTIR spectra for 44% choline hydroxide ionic liquid and choline citrate ionic liquid gel are shown in Figure 2. The peaks related to 44% choline hydroxide, i.e., O–H stretching vibration; the aliphatic C–H stretching vibration; methyl deformation; C–O stretching; and deformation of the –CH2– group were observed at 3600–3200, 2800, 1472, 1077, and 951 cm^−1^ in the case of choline citrate additional peaks of stretching vibrations (C=O) and antisymmetric stretching (COO^-^) at 1720 and 1572 cm^−1^ [23,24,25].

### 3.3. Tooth Color Analysis

#### 3.3.1. Shade Matching with Vitapan Shade Guide

**Figure 3:**
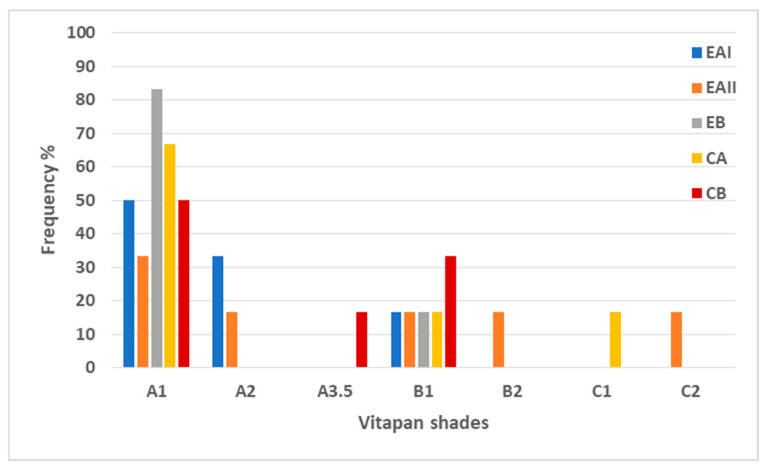
Frequency% of the Vitapan shades across the groups. All the tooth samples treated with synthesized EB (choline citrate gel) showed A1 shades in comparison to the CA-treated tooth samples giving a range of shades, i.e., A1, B1, and C1.

The results of the Vitapan shade guide were analyzed with Fisher’s exact test. The unstained tooth samples in each group had an A1 shade, and the stained tooth samples had an A4 shade. A clustered bar chart shown in Figure 3 demonstrates the frequency percentage of the Vitapan shades across five groups. The A1 shade was the most frequent shade detected amongst the five groups. The highest frequency percentage of the A1 shade was noted in EB, i.e., 83.3%, and lowest in EAII, i.e., 33.3%. EAI and CB showed 50% and CA 66.7% of the A1 shade.

All the tooth samples treated with synthesized EB (choline citrate gel) gave A1 shades in comparison to CA-treated tooth samples giving a range of shades, i.e., A1, B1, and C1. The chi-square test for comparison revealed no significant difference among the groups (*p* > 0.05).

**Figure 4:**
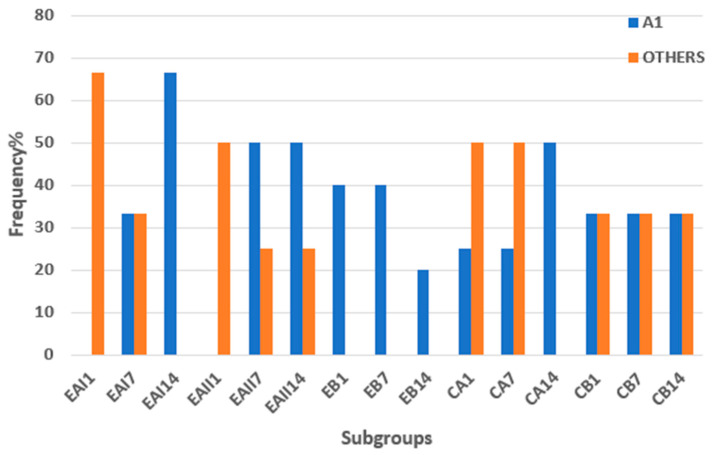
Frequency% of A1 shades across subgroups. EB (choline citrate gel) showed the A1 shade after 7 h (1 night), 49 h (7 nights), and 98 h (14 nights) of treatment duration as compared to the CA (commercial at-home 16% carbamide peroxide gel). The A1 shade was achieved earlier, i.e., after 7 h and 49 h of treatment with choline citrate gel (EB), in comparison to the commercial at-home 16% carbamide peroxide (CA) that showed the A1 shade after 98 h of application.

For a comprehensive analysis, the Vitapan shade guide results were broadly categorized as A1 and others. The bar graph in Figure 4 displays the frequency percentage of A1 shades as compared to the other shades in five groups across the treatment duration subgroups. The bar graph in Figure 4 illustrates that all the synthesized EB (choline citrate gel)-treated tooth samples showed an A1 shade after 7 h (1 night), 49 h (7 nights), and 98 h (14 nights) of treatment duration as compared to the CA (commercial at-home 16% carbamide peroxide gel). The results also suggest that the A1 shade was achieved earlier, i.e., after 7 h and 49 h of treatment with choline citrate gel (EB), in comparison to the commercial at-home 16% carbamide peroxide (CA) that showed the A1 shade after 98 h of application.

#### 3.3.2. Digital Colorimetry

The mean ΔE values of the tooth samples for five groups after 7 h (1 night), 49 h (7 nights), and 98 h (14 nights) of treatment are tabulated in Table 3. The values obtained via digital colorimetry were analyzed with two-way independent ANOVA. The pairwise comparisons among the groups revealed no significant difference (*p* > 0.05) in the ΔE values of the tooth samples.

**Figure 5:**
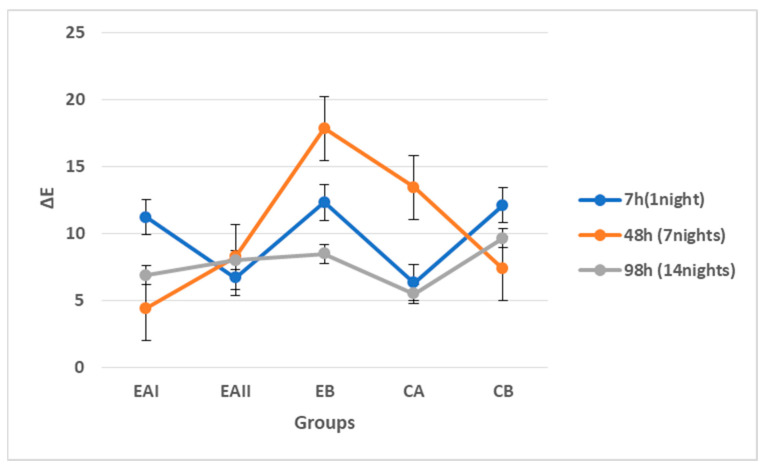
Trend of ΔE for the treatment duration across five groups. At 7 h, EAI, EB, and CB showed ΔE values in the same range, i.e., ΔE > 10 with EAII and CA, showing ΔE values in the same range, i.e., ΔE < 10. At 49 h, EB and CA showed ΔE values in the same range, i.e., ΔE > 10, with EAI, EAII, and CB showing ΔE values in the same range, i.e., ΔE < 10. At 98 h, all the five groups showed ΔE values in the same range, i.e., ΔE < 10.

Figure 5 gives a graph showing the trend of ΔE for 7 h (1 night), 49 h (7 nights), and 98 h (14 nights) of treatment duration across five groups. There was no statistically significant difference (*p* > 0.05) in the ΔE values between 7 h (1 night), 49 h (7 nights), and 98 h (14 nights) duration of treatment subgroups.

The line graph in Figure 5 shows that, at 7 h (1 night), of treatment duration, EAI, EB, and CB showed ΔE values in the same range, i.e., ΔE > 10, with EAII and CA showing ΔE values in the same range, i.e., ΔE < 10. At 49 h (7 nights) of treatment duration, EB and CA showed ΔE values in the same range, i.e., ΔE > 10, with EAI, EAII, and CB showing ΔE values in the same range, i.e., ΔE < 10. A treatment duration of 98 h (14 nights) showed ΔE values in the same range across the five groups of ΔE < 10. Overall, the data shown in the graphs represent nonlinear in behavior.

**Figure 6:**
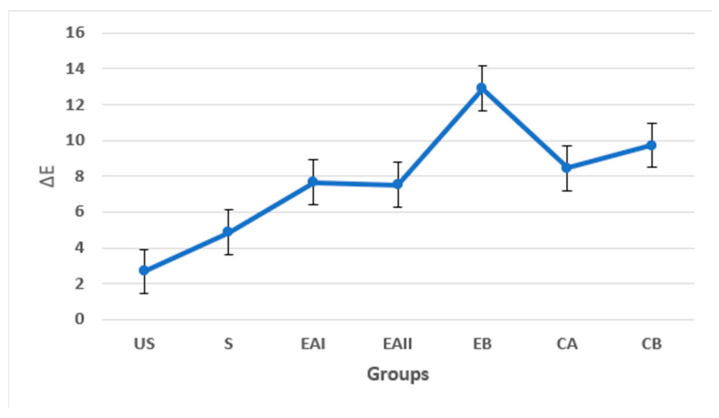
Trend of ΔE across groups in comparison to the Unstained (US) and Stained (S) tooth samples. EB exhibited ΔE values in a different range of ΔE > 10 as compared to all the other groups.

Figure 6 shows the trend of the ΔE values of the tooth samples amongst the five groups concerning the mean ΔE values of the Unstained (US) and Stained (S) tooth samples. This trend line portrays that group EB exhibited ΔE values in a different range of ΔE > 10 as compared to all the other groups, which showed ΔE values in the same range of ΔE < 10.

### 3.4. Microhardness

The mean microhardness values (Vickers Hardness Number—VHN) and standard deviations of the tooth samples for five groups are depicted in Table 4. The pairwise comparisons amongst the groups revealed no significant difference (*p* > 0.05) in the microhardness values of the tooth samples.

**Figure 7:**
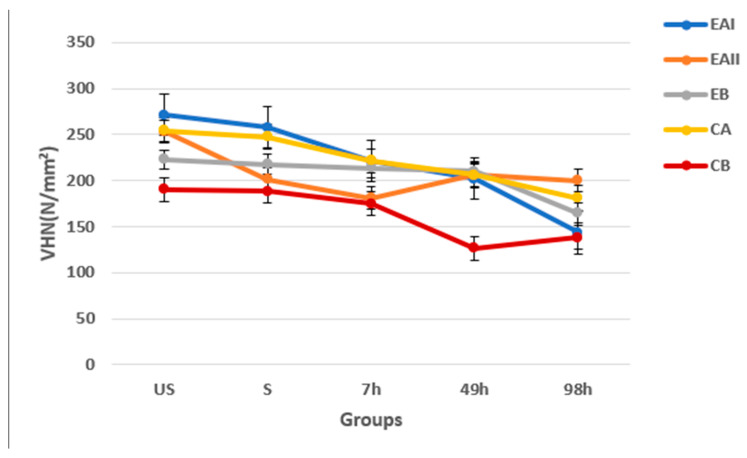
Trend of microhardness across groups in comparison to the Unstained (US) and Stained (S) tooth samples. A progressive decrease in microhardness across the five groups while moving from the Unstained tooth sample to 98 h treatment in duration.

Figure 7 shows the trend of microhardness values of the tooth samples amongst the five groups concerning the mean microhardness values of the unstained and stained (without any treatment) tooth samples.

This trend shows a progressive decrease in microhardness across the five groups while moving from the unstained tooth sample to 98 h (14 nights) treatment in duration. However, an increase of microhardness in group EAII at 49 h (7 nights) and CB at 98 h (14 nights) was observed, which was different from the trend shown otherwise.

### 3.5. Scanning Electron Microscopy

**Figure 8:**
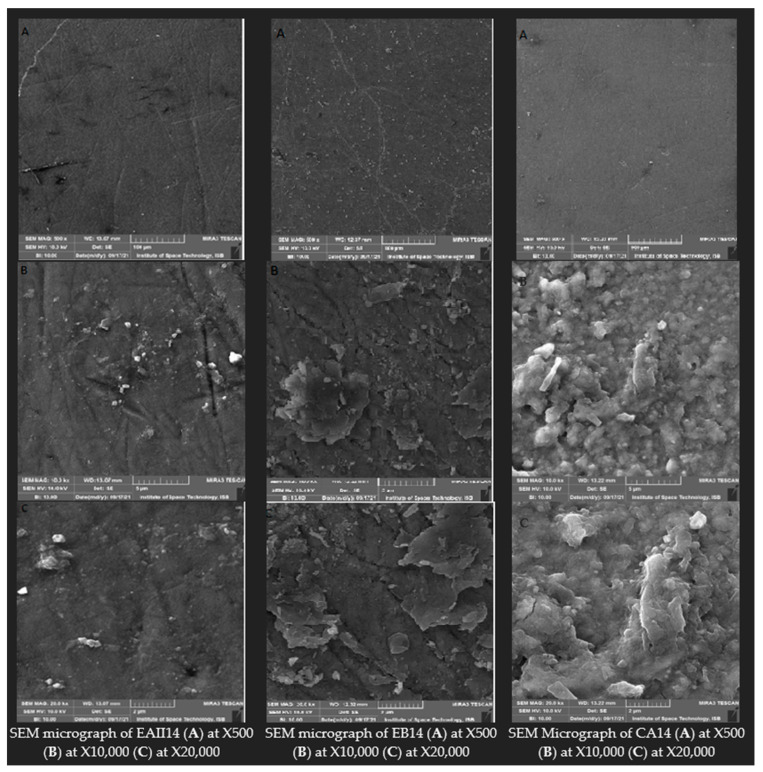
SEM images of EAII14, EB14, and CA14 at 500, 10,000, and 20,000 magnifications.

The SEM images of EAII14, EB14, and CA14 at X500, X10,000, and X20,000 are depicted in Figure 8. The EAII14 micrograph shows a typical smooth enamel intact surface with indentations and deposits. The EB14 micrograph shows flakes in the shape structure with an intact enamel surface, indentations, and deposits. In the case of CA14, the micrograph shows the etching effect of the rough surface, along with indentations and deposits.

### 3.6. Energy Dispersive X-ray Spectroscopy

**Figure 9:**
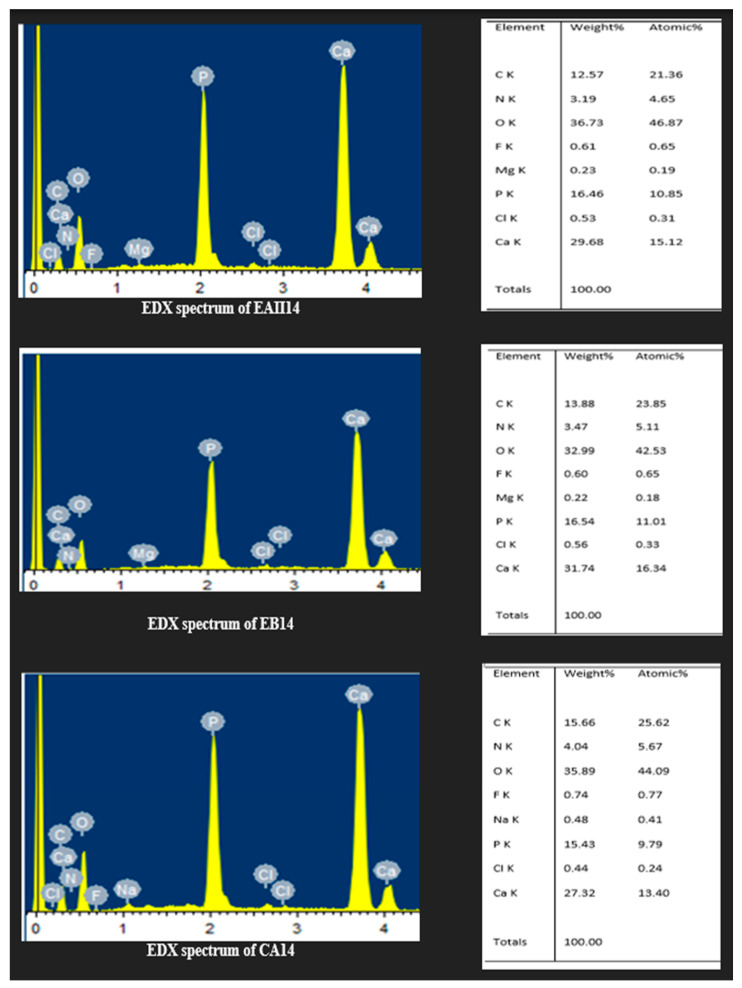
EDX analysis of the tooth samples of the EAII14, EB14, and CA14 groups.

An elemental analysis by EDX of EAII14, EB14, and CA14 is depicted in Figure 9. The Ca/P ratios of EAII14, EB14, and CA14 were 1.8, 1.91, and 1.77, respectively.

### 3.7. Optical Profilometry

For optical profilometry, four subgroups were selected: EAI14, EAII14, EB14, and CA14 of 14 nights (98 h) of treatment. The roughness average (Ra) values of the tooth samples, along with top view and 3D topographical images, were obtained. Table 5 enlists the Ra values. The comparison of the roughness average of the tooth samples in the tested four subgroups with one-way ANOVA revealed no significant difference (*p* > 0.05).

**Figure 10:**
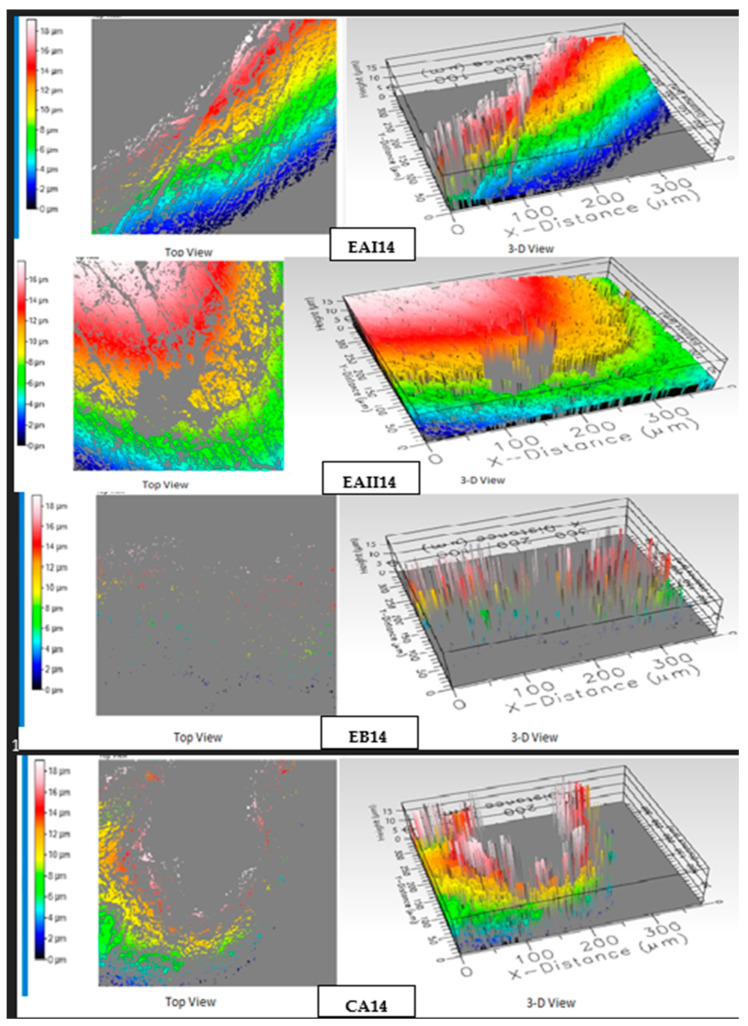
Top and 3D surface topography of the EAI14, EAII14, EB14, and CA14 groups.

The profilometry images (top view and 3D view) for EAI14, EAII14, EB14, and CA14 are shown in Figure 10.

## 4. Discussion

Tooth bleaching is regarded as a cosmetic dental treatment to lighten the color of teeth. It is indicated for generalized or localized dental stains due to aging, fluorosis, smoking, and dietary stains [8]. Some dental procedures may also require tooth bleaching after the orthodontic treatment [26]. Noninvasive cosmetic treatment for teeth requires the use of a biosafe material. There are reports of the damaging effects of peroxide bleaching agents on oral tissues [5,9,10].

The introduction of organic, environment-friendly materials and procedures is endorsed nowadays. There are apprehensions about free radicals as they increase oxidative stress, causing infectious diseases such as kidney disease and neurodegenerative disorders [27,28]. Considering these rising issues, this research took the initiative to introduce the use of bio-friendly, antiaging materials in cosmetic dentistry with a safer impact at the local and global levels.

The active ingredient used in tooth bleaching gels is hydrogen peroxide incorporated in varying concentrations. Hydrogen peroxide in its active form is incorporated in the gels that are used for in-office tooth bleaching. On the other hand, carbamide peroxide is incorporated in dentist-prescribed at-home tooth bleaching kits, in which hydrogen peroxide is present in its inactive form [26]. This is to ensure the safety of the patient while using this ingredient, known for its corrosive and bio-hazardous role [8].

In this context, research on a safer, biocompatible, cost-effective active bleaching agent with the potential to replace hydrogen peroxide is underway. Certain agents other than hydrogen peroxide are used in over-the-counter bleaching products; however, they are yet not approved to be used in-office or dentist-prescribed at-home tooth bleaching procedures.

In this study, choline hydroxide in two concentrations, i.e., 22% and 44%, to analyze its results at the lower and higher ends, was made. A second gel that incorporated choline citrate was also made. Citric acid was incorporated in choline hydroxide to attain a neutral pH of the synthesized gel. It is attributed to possessing tooth bleaching action as well [29,30].

The pH of the oral cavity is maintained within the range of 6.2 to 7.6 by the rearrangements of electrolytes in the composition of saliva [31]. However, as this pH falls below 5.2, the demineralization of the tooth enamel and root resorption process is hastened [32,33]. Consequently, subjecting the oral structures to low or high pH for a prolonged duration may cause adverse effects, of which the most reported one is the erosion of enamel [34].

Various at-home tooth bleaching products available vary in their pH, ranging from 4 to 7.5 [35]. Another study on twenty-six commercially available tooth bleaching products reported this range from 3.67 to 11.13; this matches with the pH range found in the current study, i.e., 12.4 to 5.53 [36]. The mean pH of the four gels and one ionic liquid in the study came out to be 8.70 ± 2.63, which has been reported as 6.83 ± 1.27 and, in another study, as 8.22 ± 2.0 [35,36].

It has been reported that an increased concentration of peroxides in the tooth bleaching gel shifts the pH towards the acidic side [33]. Studies on peroxide-based tooth bleaching gels reported mean pH of 6.48 ± 1.27 and 5.56 ± 1.64; this value is in close approximation to the pH of the at-home 16% carbamide peroxide gel used in the study, i.e., 5.53 ± 0.20 [36,37]. Hence, peroxide-based products are usually towards the acidic side of the pH line that could be due to the presence of citric acid [38].

In the literature, 44% choline hydroxide is reported to be a basic liquid with a pH of 12.5, which is similar to the value obtained in this study, i.e., 12.4 [36,37]. However, 44% choline hydroxide gel shows a 11.2 pH, which suggests the presence of starch, which is a weak acid with a pH range of 4 to 5 [39,40]. In the case of 22% choline hydroxide gel, the choline–hydroxide gel was diluted, which may have contributed to its lower basic potential.

Spectroscopic analyses of choline hydroxide in various studies have revealed the presence of O–H, C–H, and C–N groups [41]. All these peaks were detected in the FTIR spectra of 44% choline hydroxide liquid, 22% choline hydroxide gels, and 44% choline hydroxide gels. The presence of these groups confirms that two synthesized gels were choline hydroxide-based. Choline citrate gel contains choline hydroxide with citrate in its composition. The FTIR spectra of this gel contain an additional carboxylic acid, i.e., the COOH group, along with the O–H, C–H, and C–N groups [23,39].

Several different color analysis methods are employed in tooth bleaching studies. However, the acceptance criteria laid down by the American Dental Association (ADA) for research on at-home tooth bleaching involves the use of subjective and objective methods for the tooth color analysis. The subjective color analysis requires the use of value-oriented shade guides, and for an objective assessment, an electronic color measurement device is to be used [42].

The most widely used shade guide in dentistry is the Vitapan classical shade guide (Eachempati et al. 2018). It was also used in the current study, and it helped in standardizing the color measurement process together with showing consistent results. It allows the dentist and patient to discuss and visualize in-office or at-home tooth bleaching while using the same terminologies [43,44].

This study showed that the bleaching treatment did cause an improvement in the shade of stained tooth samples in the five groups. Group EB (choline citrate gel group), however, outperformed CA (at-home carbamide peroxide gel group) and CB (deionized water group). All the tooth samples treated with synthesized choline citrate gel showed A1 and B1 shades in comparison to the commercial at-home carbamide peroxide gel-treated tooth samples showing a range of shades, i.e., A2, B1, and C1.

All the synthesized choline citrate gel-treated tooth samples showed the A1 shade after 7 h (1 night), 49 h (7 nights), and 98 h (14 nights) in treatment duration as compared to the commercial at-home 16% carbamide peroxide gel. Hence, the choline citrate gel in comparison to the commercial at-home 16% carbamide peroxide was better; therefore, the A1 shade was achieved earlier, i.e., after 7 h and 49 h of treatment, while that showed the A1 shade after 98 h of application.

In general, the Vitapan shade guide results suggested that the shade improvement of stained teeth with the synthesized tooth bleaching gels (22% choline hydroxide, 44% choline hydroxide, and choline citrate gel) does occur. The comparison amongst the groups and subgroups was insignificant (*p >* 0.05), which can be attributed to the small sample size selected for the study. However, a closer analysis establishes that the synthesized choline citrate is a promising tooth bleaching gel among the five tested groups in the current study.

The assessment of color and its accurate reproduction are one of the two most important and challenging aspects of dentistry so far [45]. It is for this reason that, for objective and accurate clinical tooth shade selection, electronic shade matching devices are used. In the current study, for an objective analysis of the ΔE values obtained, a standard perception range given by Zachary Schuessle was followed [46].

It was observed that EAI (22% choline hydroxide gel group), EAII (44% choline hydroxide gel group), CA (commercial at-home carbamide peroxide gel group), and CB (deionized water group) showed ΔE values in the same range, which is clinically perceptible (2–10 ΔE) at a glance. However, EB (choline citrate gel group) showed the highest value of ΔE, producing a color change that is perceptibly similar to the opposite color. The trend line also confirmed that group EB (choline citrate gel group) exhibited ΔE values in a different range of ΔE > 10 as compared to all the other groups, which showed ΔE values in the same range of ΔE < 10.

Choline citrate gel and positive control commercially at-home carbamide peroxide gel had a similar ΔE range during 7 h, 49 h, and 98 h of application. It was observed that synthesized 22% and 44% choline hydroxide gels also produced noticeable improvements in tooth color after 98 h duration of treatment.

A comparative study on the in-office and at-home tooth bleaching with hydrogen peroxide and carbamide peroxide showed ΔE < 10, which increased as these two treatments were combined [47]. A study done to compare the effect of repeated bleaching with 16% carbamide peroxide revealed that a change in ΔE causes a lightening in the color of treated tooth samples [48,49]. A similar trend was observed in our study.

Overall, combining the results of the Vitapan shade guide and digital colorimetry, it is suggested that improvement in the color of stained teeth with the synthesized tooth bleaching gels (22% choline hydroxide, 44% choline hydroxide, and choline citrate gel) occurred.

To assess the alteration in the mechanical characteristics of enamel after the bleaching treatment, microhardness tests were conducted [50]. The mean microhardness values depicted for the five groups showed that the effect of three experimental gels, i.e., 44% choline hydroxide, 22% choline hydroxide, and choline citrate, on the microhardness of the tooth samples was similar to that of the positive control, which comprised commercial at-home 16% carbamide peroxide gel.

The trend of microhardness displayed in the five groups in comparison to the Unstained and Stained tooth samples showed a progressive decrease. The VHN of the Unstained tooth samples was the highest. It decreased as it was stained, which further kept on decreasing as it was bleached for 7 h (1 night), 49 h (7 nights), and 98 h (14 nights). The statistically significant difference (*p* < 0.05) in the microhardness values between 7 h (1 night), 49 h (7 nights), and 98 h in duration of the treatment subgroups points towards the notion that the duration of treatment does affect the enamel microhardness. These results conform with numerous studies done to assess the effects of peroxide-based tooth bleaching gels on the mechanical characteristics of tooth enamel [51,52,53].

An increase of microhardness in the 44% choline hydroxide gel group (EAII) at 49 h (7 nights) and deionized water group (CB) at 98 h (14 nights) was observed, which was different from the trend depicted otherwise. This discrepancy in the trend might be attributed to the design of the methodology for this test, in which different samples were used for each category, i.e., Unstained and Stained 7 h, 49 h, and 98 h.

The Vickers Hardness Number of the tooth enamel was 250 to 300 N/mm², making it a hard and brittle structure [54]. Some studies reported no effect on the enamel microhardness after bleaching; however, others showed a decrease [55,56,57]. The reduction in microhardness of tooth samples when they were bleached can be attributed to two reasons. The first reason may be attributed to the pH of tooth bleaching gels, which usually does not correlates to the normal pH range of the oral cavity, causing demineralization of the enamel and decreasing the calcium phosphorus ratio [58,59]. The second one can be attributed to the mechanism of action of tooth bleaching gels that involves the oxidation of stain molecules causing micromorphological alterations in the enamel that results in decreasing its microhardness [59,60].

The micrograph in Figure 8 shows the tooth samples treated with 44% choline hydroxide gel (EAII14) and choline citrate gel (EB14), and the commercial at-home 16% carbamide peroxide gel (CA14) for 98 h (14 nights) shows a typical appearance of enamel with deep indentations and flake shapes (only in EB14) and deposits. The deep indentation, flake shapes debris, and deposits on the tooth surfaces are related to tooth cutting and polishing. At higher magnifications, the micrographs of EAII14 and EB14 showed intact surfaces while CA14 showed eroded surfaces with an etching effect. In other SEM studies, tooth bleaching with hydrogen peroxide and carbamide peroxide has shown evidence of erosions, increases in porosities, and shallow indentations [61,62,63].

The Ca/P ratio of EAII14, EB14, and CA14 was 1.8, 1.91, and 1.77, respectively, which is higher than the reported values, i.e., 1.67, for bovine tooth enamel [64]. This rise in the Ca/P ratio was attributed to the removal of the organic matrix (stains) of the tooth structure. In comparison, CA14 showed a lower Ca/P ratio that might be related to the dissolution of hydroxyapatite crystals, as reported in other studies [65,66]. Similarly, a decrease in the Ca/P ratio and magnesium/phosphate ratio was reported with the use of 30% hydrogen peroxide [67]. Analyzing the results of SEM with EDX together, it appears that the bleaching action by the three gels did not disturb the mineral content of the treated tooth samples to a pronounced extent.

To assess the surface profile of the tooth enamels qualitatively and quantitatively, 3D and 2D optical profilometers are used [68]. In the present study, a qualitative analysis of the surface profile was made via profilometric images of the selected area, and a quantitative analysis was acquired via computation of the roughness average of the tooth samples by using a 3D profilometer.

An increased roughness average of the tooth surface is clinically depicted as coarseness that causes plaque retention, bacterial adhesion, and the staining of teeth [69]. The roughness average values depicted the same range; hence, there was no statistically significant differences between the four tested subgroups. The lowest value was shown by the choline citrate gel subgroup, and the highest value was depicted by the 44% choline hydroxide gel subgroup. In general, the values measured in this study for bovine teeth were as high as reported in the literature, which might be the source of the tooth (human tooth) or techniques used [70,71].

The profilometric images in Figure 10 of synthesized 22% choline hydroxide, 44% choline hydroxide, and commercial at-home 16% carbamide peroxide gel-treated samples show similar rough surface topography. However, the profilometric image of synthesized choline citrate gel-treated samples demonstrated negligible alterations in the surface topography. Studies done on peroxide-based bleaching agents report an increase in surface roughness and porosity, causing significant alterations of the surface topography as viewed from the profilometric images and roughness average [70,71].

## 5. Conclusions

The synthesized novel experimental tooth bleaching gels displayed similar tooth bleaching actions without any deleterious effects on the surface characteristics and microhardness of the treated tooth samples when compared with the commercial at-home tooth bleaching gel.

## Figures and Tables

**Table 1:** Grouping scheme for the bleaching of tooth samples.

Groups	Description	Subgroups (*n* = 3)	Treatment Duration
Experimental groups (E)			
EAI	22% Choline Hydroxide gel	EAI1	7 h (1 night)
		EAI7	49 h (7 nights)
		EAI14	98 h (14 nights)
EAII	44% Choline Hydroxide gel	EAII1	7 h (1 night)
		EAII7	49 h (7 nights)
		EAII14	98 h (14 nights)
EB	Choline Citrate gel	EB1	7 h (1 night)
		EB7	49 h (7 nights)
		EB14	98 h (14 nights)
Control groups (C)			
CA	Commercial at-home carbamide peroxide gel (positive control)	CA1	7 h (1 night)
		CA7	49 h (7 nights)
		CA14	98 h (14 nights)
CB	Deionized water (negative control)	CB1	7 h (1 night)
		CB7	49 h (7 nights)
		CB14	98 h (14 nights)

**Table 2:** Mean pH and SD of parent ionic liquid and gels.

Samples	Mean pH & SD
Parent ionic liquid	
44% choline hydroxide Liquid	12.4 ± 0.15
Gels	
22% choline hydroxide gel	8.43 ± 0.51
44% choline hydroxide gel	11.2 ± 0.1
Choline citrate gel	5.86 ± 0. 05
16% at-home carbamide peroxide gel	5.53 ± 0.20
Mean pH	8.70 ± 2.63

**Table 3:** Mean ΔE values of the tooth samples.

Groups	Description	Mean ΔE and SD
Experimental groups (E)		
EAI	22% choline hydroxide gel	7.527 ± 5.66
EAII	44% choline hydroxide gel	7.663 ± 1.84
EB	Choline citrate gel	12.908 ± 6.67
Control groups (C)		
CA	Commercial at-home carbamide peroxide gel (Positive control)	8.445 ± 5.20
CB	Deionized water (Negative control)	9.722 ± 3.19

**Table 4:** Mean microhardness values of the tooth samples after treatment.

Groups	Description	Mean VHN (N/mm²) and SD
Experimental groups (E)		
EAI	22% choline hydroxide gel	188.92 ± 42.59
EAII	44% choline hydroxide gel	195.77 ± 52.58
EB	Choline citrate gel	196.29 ± 54.51
Control groups (C)		
CA	Commercial at-home carbamide peroxide gel (positive control)	202.92 ± 44.23
CB	Deionized water (negative control)	161.29 ± 58.45

**Table 5:** Roughness average of the tooth samples in the four subgroups.

Subgroups	Description	Roughness Average (µ-in)
EAI14	22% choline hydroxide	7.84
EAII14	44% choline hydroxide	10.9
EB14	Choline citrate	5.91
CA14	At-home carbamide peroxide	10.34

## Data Availability

Available on request.
